# Does a lack of physical activity explain the rheumatoid arthritis lipid profile?

**DOI:** 10.1186/s12944-017-0427-4

**Published:** 2017-02-10

**Authors:** Hiba AbouAssi, Margery A. Connelly, Lori A. Bateman, K. Noelle Tune, Janet L. Huebner, Virginia B. Kraus, Deborah A. Winegar, James D. Otvos, William E. Kraus, Kim M. Huffman

**Affiliations:** 1 Department of Medicine and Duke Molecular Physiology Institute, Duke School of Medicine, Durham, NC USA; 2 LipoScience, Inc., Laboratory Corporation of America® Holdings, Raleigh, NC USA; 3 University of North Carolina, Chapel Hill, NC USA

**Keywords:** Lipoproteins, Cardiovascular risk, Inflammation, Exercise, Arthritis

## Abstract

**Background:**

In rheumatoid arthritis (RA), cardiovascular risk is associated with paradoxical reductions in total cholesterol, low density lipoprotein-cholesterol (LDL-C), and high density lipoprotein-cholesterol (HDL-C). Concentrations of small LDL (LDL-P) and HDL (HDL-P) particles are also reduced with increased inflammation and disease activity in RA patients. Here we sought to identify which measure(s) of inflammation, disease activity and cardiometabolic risk contribute most to the RA-associated lipoprotein profile.

**Methods:**

NMR lipoprotein measurements were obtained for individuals with RA (*n* = 50) and age-, gender-, and body mass index (BMI)-matched controls (*n* = 39). Groups were compared using 39 matched pairs with 11 additional subjects used in RA only analyses. Among RA patients, relationships were determined for lipoprotein parameters with measures of disease activity, disability, pain, inflammation, body composition, insulin sensitivity and exercise. Percentage of time spent in basal activity (<1 metabolic equivalent) and exercise (≥3 metabolic equivalents) were objectively-determined.

**Results:**

Subjects with RA had fewer total and small LDL-P as well as larger LDL and HDL size (*P* < 0.05). Among RA patients, pain and disability were associated with fewer small HDL-P (*P* < 0.05), while interleukin (IL)-6, IL-18, and TNF-α were associated with LDL size (*P* < 0.05). BMI, waist circumference, abdominal visceral adiposity and insulin resistance were associated with more total and small LDL-P, fewer large HDL-P, and a reduction in HDL size (*P* < 0.05). Most similar to the RA lipoprotein profile, more basal activity (minimal physical activity) and less exercise time were associated with fewer small LDL-P and total and small HDL-P (*P* < 0.05).

**Conclusions:**

The RA-associated lipoprotein profile is associated with a lack of physical activity.

As this was a cross-sectional investigation and not an intervention and was performed from 2008–13, this study was not registered in clinicaltrials.gov.

## Background

Patients with rheumatoid arthritis (RA), a chronic inflammatory disease, have a 2- to 3-fold increased risk of cardiovascular disease (CVD) [1–4]. This has been attributed to disease-associated chronic inflammation, physical inactivity, increased adiposity, insulin resistance, and altered lipid profiles [5, 6]. Despite the increased CVD risk, RA patients often present with reduced total cholesterol, low density lipoprotein cholesterol (LDL-C) and high density lipoprotein cholesterol (HDL-C), which is opposite of the lipid profile typically observed in subjects at high risk of CVD [7–9]. Hence, the relationship between high CVD risk and lower cholesterol levels in RA patients has been referred to as the “lipid paradox.” [7–9].

Efforts to better understand the “lipid paradox” have included the measurement of lipoprotein particles via nuclear magnetic resonance spectroscopy (NMR) in patients with RA [10]. Compared to controls, RA patients have fewer small LDL (LDL-P) and small HDL (HDL-P) particles [10]. Moreover, small HDL-P are inversely associated with disease activity and coronary artery calcification in RA patients [10]. In active RA, HDL particles contain pro-inflammatory proteins such as serum amyloid A and fibrinogen which often replace anti-inflammatory and anti-oxidant molecules, which alters their function [11]. All in all, inflammation mediates alterations in HDL particle size and function that may contribute to the increased CVD observed in RA patients [12]. Furthermore, after anti-inflammatory therapy, total cholesterol, LDL-C, HDL-C, HDL-P levels increase [13]. Such increases have been attributed to reduced inflammation and cholesterol ester catabolism leading to an increase in lipoprotein cholesteryl ester levels [13]. While increased cholesterol levels are often associated with increased CVD risk, in the context of chronic inflammatory disease increased circulating cholesterol may be a reflection of the reduced inflammation and an accompanied reduction in CVD risk [13, 14].

Unfortunately, the changes observed in circulating lipids and lipoproteins in RA patients are complex as many factors besides inflammation contribute to the overall picture. For example, there are known alterations in cholesterol and lipoproteins that occur in subjects who are overweight with metabolic syndrome and/or type 2 diabetes. These include reductions in HDL-C, large HDL-P and large LDL-P and an increase in triglycerides, small LDL-P and small HDL-P, while LDL-C levels often remain in the normal range. In contrast, exercise often reverses these changes. High amounts of exercise lead to decreased small LDL-P and total LDL-P and increased large HDL-P, HDL-C as well as HDL and LDL size [15]. Patients with RA are often overweight, especially when regular physical activity is impaired by disease activity and joint damage. Therefore, disease activity, physical activity and metabolic syndrome risk factors such as body mass index (BMI), waist circumference and insulin resistance, may confound the effects of inflammation on cholesterol and lipoprotein parameters in patients with RA.

In a recent study in established and treated RA patients, we found that traditional metabolic risk factors such as excess adiposity played a more prominent role in predicting skeletal muscle insulin sensitivity than systemic inflammation or other disease-related factors.[16] However, we hypothesized that the changes observed in the “lipid paradox” are less related to metabolic syndrome risk factors and more related to lack of physical activity as a result of increased disease activity, pain and inflammation. To test this hypothesis we looked at the associations between NMR lipoprotein parameters and various measures of disease activity, inflammation, body composition, insulin sensitivity and physical activity (basal activity vs. exercise).

## Methods

### Participants and design

The study design and procedures have been previously reported [16]. Briefly, this was a cross-sectional comparison of insulin sensitivity between persons with RA and age- (+/− three years), gender-, race-, and BMI (+/− 3 kg/m^2^) -matched controls (*n* = 39 each). An additional 11 subjects with RA without a match were include in RA-only analyses. Persons with RA were either seropositive or had erosions on radiographs, met 1987 American College of Rheumatology criteria for RA [17], had no medication changes in the last three months, and were using stable doses of prednisone of 5 mg or less per day. Exclusions were known diabetes mellitus or CVD. Five persons with RA and two controls were using HMG CoA reductase inhibitors (statins); findings were not altered substantially when these persons were excluded from analyses. The study was in compliance with the Helsinki Declaration and was approved by the Duke University Medical Center Institutional Review Board (#7701).

### Assessments

Physical examinations and questionnaires assessed anthropometrics, disease activity, pain, and disability [16]. Insulin sensitivity was calculated from glucose and insulin concentrations during a frequently sampled intravenous glucose tolerance test [16]. Fasting concentrations of inflammatory cytokines were measured using ELISAs [16]. Abdominal and thigh adipose depots were measured using abdominal and thigh CT scans. Physical activity was measured using seven days of accelerometry (RT3, Stayhealthy, Inc., Monrovia, CA) [16, 18]. Data were excluded from analyses as follows: nonwear time defined as 90 consecutive minutes of zero activity data; days with less than 10 h of data; participants with less than four (10-h) days. Physical activity analyses included 41 RA participants. Basal activity was defined as less than one metabolic equivalent, the predicted amount of oxygen consumed while sitting at rest [16]. Exercise was defined as the combination of moderate, high, and very high intensity activity or greater than or equal to 3 total metabolic equivalents [16].

### Lipid and lipoprotein measurements

NMR spectra were acquired from EDTA plasma samples as previously described for the *NMR LipoProfile®* (lipoprotein particle) test at LipoScience, Inc. (now LabCorp-Raleigh, NC) using the LipoProfile-3 algorithm [19]. Mean VLDL, LDL and HDL particle sizes are weighted averages derived from the sum of the diameter of each subclass multiplied by its relative mass percentage. The NMR Profiler platform is comprised of a 9.4 T (400 MHz ^1^H frequency) spectrometer (Bruker Biospin) with an integrated fluidics sample delivery system. Total cholesterol, triglycerides, LDL-C and HDL-C were measured using standard automated methods.

### Statistical analyses

Total cholesterol and LDL-C demonstrated a normal distribution confirmed with Kolmogorov-Smirnov Goodness of Fit testing. Triglycerides, HDL-C, inflammatory markers (high sensitivity C-reactive protein [hsCRP], interleukin [IL]-1β, IL-6, IL-18, tumor necrosis factor [TNF]-α), and lipoproteins were logarithmically transformed prior to group comparisons. RA and controls (*n* = 39 each) were compared using mixed models, which accounted for the repeated measure of matched participants. Bivariate associations were assessed with Spearman correlations. Lipoprotein particle modeling was performed with general linear models. All analyses were performed in SAS (Version 9.4, Cary, NC). A priori levels of significance were *P* < 0.1 for interactions and *P* < 0.05 for all other analyses.

## Results

### Lipoprotein profile in subjects with RA

While triglycerides and HDL-C did not differ between RA and controls, both total cholesterol and LDL-C were lower in persons with RA (Table 1; *P* < 0.05 for both). For NMR-measured lipoproteins as compared to controls, RA had lower concentrations of both total LDL-P and small LDL-P, with a larger mean LDL particle size (Table 1; *P* < 0.05 for all). While total HDL-P concentrations were similar to controls, RA had a larger mean HDL particle size (Table 1; *P* < 0.05 for all).

**Table 1:** Patient demographic and clinical characteristics

	RA *n* = 50	Controls *n* = 39
Age (yrs)	55.4 ± 12.8	52.1 ± 11.4
Gender-Female	35 (70%)	27 (69%)
Race-		
Pacific Islander	1 (2%)	0 (0%)
African American	14 (28%)	12 (31%)
Caucasian	35 (70%)	27 (69%)
Body mass index (kg/m^2^)	30.5 ± 7.5	29.0 ± 5.3
Waist circumference (cm)	95.3 ± 16.7	92.1 ± 13.3
Disability (HAQ-DI)	0.7 ± 0.7	0 ± 0
Disease Activity (DAS-28)	3.0 ± 1.4	n/a
hsCRP (mg/L)	3.8 ± 4.8^†^	2.0 ± 2.8
Basal activity (% min/week)	91.2 ± 6.0	88.8 ± 6.1
Exercise (% min/week)	1.1 ± 1.5	1.5 ± 1.3
Total cholesterol (mg/dl)	176.6 ± 30.7^†^	185.9 ± 32.3
LDL-cholesterol (mg/dl)	105.0 ± 24.7^†^	117.2 ± 26.4
HDL-cholesterol (mg/dl)	53.7 ± 1.3	49.2 ± 1.3
Triglycerides (mg/dl)	93.6 ± 1.6	96.6 ± 1.5
LDL Particles – Total (nmol/L)	965.1 ± 1.3^†^	1117.6 ± 1.4
Large LDL Particles (nmol/L)	363.0 ± 1.8	363.1 ± 1.6
Small LDL Particles (nmol/L)	386.2 ± 2.1^†^	521.2 ± 2.0
LDL Particle Size (nm)	20.9 ± 1.0^†^	20.7 ± 1.0
HDL Particles – Total (μmol/L)	29.1 ± 1.2	29.3 ± 1.2
Large HDL Particles (μmol/L)	4.9 ± 1.8	3.9 ± 1.9
Medium HDL Particles (μmol/L)	8.5 ± 1.6	8.8 ± 1.9
Small HDL Particles (μmol/L)	13.8 ± 1.4	13.9 ± 1.5
HDL Particle Size (nm)	9.3 ± 1.1^†^	9.0 ± 1.1

Data are presented as mean ± SD, geometric mean ± SD for nonnormally distributed variables, or frequency (percent)

^†^Significantly different (*P* < 0.05) as compared to controls using mixed models

*HAQ-DI* Health Assessment Questionnaire Disability Index, *DAS*_*ESR*_ *-28* Disease activity score with 28 joint count using erythrocyte sedimentation rate, *hsCRP* High sensitivity C-reactive protein, *LDL* Low density lipoprotein, *HDL* High density lipoprotein, *RA* Rheumatoid arthritis

### Lipoprotein particle associations with inflammation, pain, and disability in RA

In persons with RA, greater concentrations of the inflammatory cytokines, IL-6, IL-18, and TNF-α, were associated with reduced LDL particle size, (*r* = −0.31; *r* = −0.33; *r* = −0.28; *P* < 0.05 for all). Medium HDL-P had an inverse association with TNF-α (*r* = −0.31; *P* < 0.05). Inflammatory cytokines were not significantly related to small HDL-P; although increased pain and disability were associated with fewer small HDL-P (*r* = −0.42; *r* = −0.37; *P* < 0.05 for both).

### Lipoprotein associations with body composition and insulin action in RA

In persons with RA, lipoproteins and lipoprotein cholesterol levels were associated with a number of measures of cardiometabolic risk (Table 2). For LDL, concentrations of both total LDL-P and small LDL-P were associated with greater BMI, waist circumference, abdominal visceral adiposity, homeostasis model assessment of insulin resistance [HOMA-IR], and fasting insulin (Table 2; *P* < 0.05 for all). LDL-size was associated with insulin sensitivity and inversely associated with HOMA-IR and fasting insulin (Table 2; *P* < 0.05 for all). For HDL, total HDL-P was associated with insulin sensitivity while small HDL-P was associated with waist circumference (Table 2; *P* < 0.05 for both). Large HDL-P and HDL-size were inversely correlated with BMI, waist circumference, abdominal visceral adiposity, HOMA-IR and fasting insulin (Table 2; *P* < 0.05 for all). Medium HDL-P was inversely associated with fasting insulin. LDL-C was associated with BMI, abdominal visceral adiposity and fasting insulin while HDL-C was associated with insulin sensitivity and inversely associated with waist circumference, HOMA-IR and fasting insulin (Table 2; *P* < 0.05 for all). None of the lipoprotein or lipid parameters were associated with abdominal subcutaneous adiposity.

**Table 2:** Relationships between lipoprotein parameters and various measures in persons with RA (*n* = 50)

	Total LDL-P	Large LDL-P	Small LDL-P	LDL Size	LDL-C	Total HDL-P	Large HDL-P	Medium HDL-P	Small HDL-P	HDL Size	HDL-C
Disease Activity											
Disability (HAQ-DI)	−0.10	0.11	−0.19	0.10	−0.10	−0.25	0.10	−0.02	**−0.37** ^†^	0.20	−0.10
Pain (VAS) (mm)	−0.15	−0.06	−0.13	−0.06	−0.07	**−0.32** ^†^	−0.03	0.02	**−0.42** ^**‡**^	0.11	−0.20
Disease activity (DAS_ESR_-28)	0.00	0.08	0.05	−0.04	−0.03	−0.20	−0.02	0.03	−0.26	0.06	−0.19
ESR (mm/h)	−0.08	−0.03	0.06	−0.10	−0.13	−0.02	−0.05	0.12	−0.08	−0.01	−0.09
hsCRP (mg/L)	−0.05	−0.14	0.14	−0.24	−0.04	−0.22	−0.12	−0.04	−0.07	−0.08	−0.18
IL-1β (pg/ml)	−0.15	−0.14	0.00	−0.12	−0.02	−0.02	−0.11	0.19	−0.15	−0.04	−0.00
IL-6 (pg/ml)	−0.07	−0.17	0.09	**−0.31** ^†^	−0.27	−0.12	−0.06	−0.07	0.01	−0.12	−0.21
IL-18 (pg/ml)	0.16	−0.23	0.26	**−0.33** ^†^	−0.23	−0.05	−0.26	−0.08	0.19	−0.26	−0.03
TNF-α (pg/ml)	0.26	−0.07	0.23	**−0.28** ^†^	**−0.30** ^†^	−0.26	−0.23	**−0.31** ^†^	0.14	−0.24	−0.04
Body Composition											
Body mass index (kg/m^2^)	**0.37** ^†^	0.01	**0.33** ^†^	−0.08	**0.32** ^†^	−0.14	**−0.38** ^†^	−0.23	0.24	**−0.32** ^†^	−0.22
Waist circumference (cm)	**0.39** ^†^	−0.03	**0.37** ^†^	−0.14	0.23	−0.13	**−0.44** ^**‡**^	−0.22	**0.29** ^†^	**−0.42** ^**‡**^	**−0.29** ^†^
Abdominal visceral adiposity (cm^2^)	**0.47** ^**‡**^	0.13	**0.32** ^†^	−0.07	**0.38** ^†^	−0.04	**−0.41** ^**‡**^	−0.05	0.21	**−0.39** ^†^	−0.17
Abdominal subcutaneous adiposity (cm^2^)	0.20	0.01	0.16	0.05	0.17	−0.07	−0.24	−0.14	0.14	−0.20	−0.08
Insulin Action											
Insulin sensitivity (×10^−5^ min^1^/[pmol/L])	−0.28	0.17	−0.30	**0.32** ^†^	0.18	**0.39** ^†^	0.25	0.24	0.12	0.20	**0.31** ^†^
HOMA-IR	**0.37** ^†^	−0.26	**0.53** ^**‡**^	**−0.53** ^**‡**^	0.06	−0.22	**−0.54** ^**‡**^	−0.24	0.24	**−0.53** ^**‡**^	**−0.51** ^**‡**^
Fasting insulin (mU/L)	**0.34** ^†^	−0.25	**0.43** ^**‡**^	**−0.45** ^**‡**^	**0.30** ^†^	−0.29	**−0.48** ^**‡**^	**−0.31** ^†^	0.18	**−0.46** ^**‡**^	**−0.47** ^**‡**^
Physical Activity (*n* = 41)											
Basal activity (% min)	−0.29	0.19	**−0.48** ^**‡**^	**0.33** ^†^	−0.12	**−0.31** ^†^	**0.37** ^†^	−0.02	**−0.52** ^**‡**^	**0.43** ^**‡**^	0.23
Exercise (% min)	0.18	−0.24	**0.41** ^†^	**−0.32** ^†^	−0.02	0.22	**−0.37** ^†^	0.05	**0.37** ^†^	**−0.43** ^**‡**^	−0.30

*HAQ-DI* Health Assessment Questionnaire Disability Index, *VAS* visual analogue scale, *DAS*_*ESR*_ *-28* Disease activity score with 28 joint count using erythrocyte sedimentation rate (ESR), *hsCRP* High sensitivity C-reactive protein, *HOMA-IR* Homeostatic model assessment of insulin resistance, *LDL-P* Low density lipoprotein particles, *LDL-C* Low density lipoprotein cholesterol, *HDL-P* High density lipoprotein particles, *HDL-C* High density lipoprotein cholesterol, *RA* Rheumatoid arthritis

Data are shown as Spearman correlation coefficients (r). ^†^0.005 < *P* < 0.05 ^‡^ *P* ≤ 0.005

### Lipoprotein associations with physical activity in RA

**Fig. 1:**
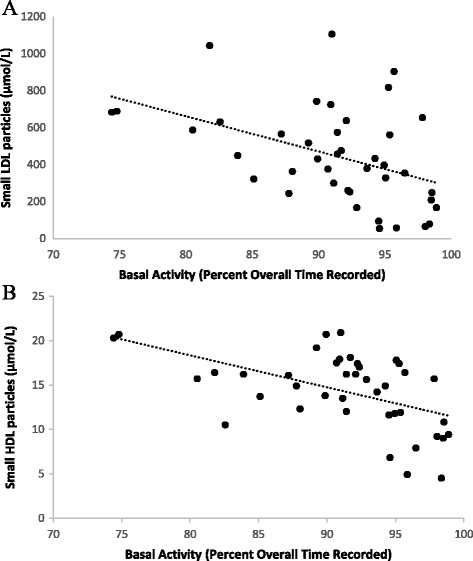
Relationship between physical activity and RA-associated lipoprotein particles. **a** Basal activity and small LDL-particles. **b** Basal activity and small HDL-particles. Physical activity was measured with seven days of accelerometry. Basal activity was defined as physical activity expending less than one metabolic equivalent. Lipoprotein subclasses were measured by NMR

Since HDL profiles appeared more related to pain and disability than disease activity or inflammation, we hypothesized that in RA, HDL profiles might be impacted by lack of physical activity. In fact, more basal activity time (minimal physical activity) was associated with fewer total HDL-P and small HDL-P (Fig. 1), more large HDL-P, and larger mean HDL-size (Table 2; *P* < 0.05 for all). On the other hand, more exercise time was associated with a profile that shifted toward more small HDL-P, fewer large HDL-P, and reduced mean HDL-size (Table 2; *P* < 0.05 for all). Further, the relationship between basal activity and small HDL-P was independent of exercise time and together, both accounted for 29% of the variance in small HDL-P (model *P* < 0.002; P_basal activity time_ < 0.005; P_exercise time_ < 0.28).

For LDL, more basal activity and less exercise time were associated with fewer small LDL-P (Fig. 1 and Table 2; *P* < 0.05 for both) and a larger mean LDL-size (Table 2; *P* < 0.05 for both). More basal activity time remained independently and inversely related to small LDL-P concentrations even when controlling for exercise time (model *P* < 0.01; model *R* ^*2*^ = 0.23; P_basal activity time_ < 0.01; P_exercise time_ = 0.37).

To determine whether the effects of minimal physical activity and exercise time on lipoproteins were impacted by age, gender, inflammation and adiposity, we performed adjusted linear regression analyses. Analyses targeted small LDL-P and small HDL-P because these were most strongly related to the physical activity measures. Similarly, basal activity or minimal physical activity time was included as the physical activity measure because it had shown stronger bivariate relationships. Basal activity was inversely associated with small HDL-P (*P* = 0.001) and this association remained significant and unattenuated even after adjusting for age, gender, disability, pain, TNFα, IL-6, BMI and abdominal visceral adiposity (*P* < 0.05) (Table 3). Pain showed a significant inverse association with small HDL-P in a model that contained basal activity, age, gender and disability (*P* < 0.05) (Table 3). However, the association of pain with small HDL-P was attenuated after further adjustment for TNFα, IL-6, BMI and abdominal visceral adiposity (*P* = 0.15). None of the other parameters showed a significant association with small HDL-P (Table 3). Basal activity was also inversely associated with small LDL-P (*P* = 0.002) and this association remained significant and unattenuated even after adjusting for age, gender, disability, pain, TNFα, IL-6, BMI and abdominal visceral adiposity (*P* < 0.05) (Table 4). Age and gender were also inversely associated with small LDL-P after adjusting for age, gender, disability, pain, TNFα, IL-6, BMI and abdominal visceral adiposity (*P* < 0.05). However, these associations were not consistently significant in every model tested (Table 4).

**Table 3:** Multivariable linear regression analysis demonstrating relationship of basal activity with small HDL-P

Small HDL-P (Log μmol/L)	Model 1		Model 2		Model 3		Model 4		Model 5		Model 6	
	β	*p* value	β	*p* value	β	*p* value	β	*p* value	β	*p* value	β	*p* value
Basal activity (% min/week)	−0.013	0.001	−0.013	0.003	−0.011	0.01	−0.012	0.004	−0.012	0.005	−0.009	0.046
Age (yrs)			−0.002	0.37	−0.003	0.10	−0.001	0.44	−0.003	0.11	−0.004	0.07
Gender			−0.020	0.71	−0.064	0.27	−0.008	0.88	−0.037	0.50	−0.055	0.39
Disability (HAQ-DI)					0.016	0.74					−0.007	0.90
Pain (VAS)					−0.002	0.03					−0.002	0.15
TNFα (pg/ml)							0.001	0.21			0.0007	0.38
IL-6 (pg/ml)							−0.002	0.33			0.0003	0.86
BMI (kg/m^2^)									0.006	0.12	0.006	0.16
Visceral adiposity (cm^2^)									0.0003	0.48	0.0003	0.44

β: Partial regression coefficient

Model 1 = unadjusted linear regression model

Model 2 = adjusted for age and gender (Parameter estimates are for women; men = 0)

Model 3 = adjusted for age, gender, disability and pain

Model 4 = adjusted for age, gender, TNFα and IL-6

Model 5 = adjusted for age, gender, BMI and visceral adiposity

Model 6 = adjusted for age, gender, disability, pain, TNFα, IL-6, BMI and visceral adiposity

**Table 4:** Multivariable linear regression analysis demonstrating relationship of basal activity with small LDL-P

Small LDL-P (Log μmol/L)	Model 1		Model 2		Model 3		Model 4		Model 5		Model 6	
	β	*p* value	β	*p* value	β	*p* value	β	*p* value	β	*p* value	β	*p* value
Basal activity (% min/week)	−0.026	0.002	−0.023	0.01	−0.022	0.02	−0.023	0.01	−0.021	0.02	−0.020	0.03
Age (yrs)			−0.007	0.06	−0.010	0.02	−0.007	0.04	−0.008	0.04	−0.011	0.01
Gender			−0.172	0.12	−0.257	0.04	−0.185	0.10	−0.217	0.07	−0.376	0.01
Disability (HAQ-DI)					−0.012	0.90					0.055	0.66
Pain (VAS)					−0.002	0.24					−0.004	0.13
TNFα (pg/ml)							−0.001	0.69			−0.002	0.18
IL-6 (pg/ml)							−0.005	0.14			−0.006	0.15
BMI (kg/m^2^)									0.013	0.10	0.013	0.13
Visceral adiposity (cm^2^)									0.0001	0.95	−0.001	0.40

β: Partial regression coefficient

Model 1 = unadjusted linear regression model

Model 2 = adjusted for age and gender (Parameter estimates are for women; men = 0)

Model 3 = adjusted for age, gender, disability and pain

Model 4 = adjusted for age, gender, TNFα and IL-6

Model 5 = adjusted for age, gender, BMI and visceral adiposity

Model 6 = adjusted for age, gender, disability, pain, TNFα, IL-6, BMI and visceral adiposity

## Discussion

Similar to prior reports, we found that compared to controls, subjects with RA had lower NMR-measured concentrations of total and small LDL-particles, yet larger LDL- and HDL-particle sizes [10, 20]. There were trends toward fewer small particles and/or more large particles in both LDL and HDL lipoprotein classes. Here, we expanded the current knowledge regarding RA lipoprotein profiles and demonstrate associations for each of physical activity, inflammation, and traditional cardiometabolic risk factors. Most similar to the RA lipoprotein profile, more time spent in basal activity (minimal physical activity) and less spent in exercise were associated with fewer small LDL and small HDL particles.

To our knowledge, this is the first report suggesting that the unfavorable HDL profile (fewer small particles) associated with RA may result in large part from minimal physical activity. Among those with RA, basal activity was associated with less small HDL-, fewer total HDL-particles, and an increased mean HDL-size. Additionally, time spent in exercise had the inverse effect on HDL profiles. Further, fewer small HDL concentrations were associated with pain and disability, while there were no HDL associations with cytokines. In persons without RA, exercise is known to exert beneficial effects on HDL-particles [15, 21, 22]. However, these exercise effects are due primarily to increases in large and medium, rather than small, HDL-particles [15, 23]. Thus, our observed effects of inactivity and exercise on small HDL may be unique to those with active RA—and perhaps other inflammatory diseases.

NMR-measured HDL parameters in persons with RA were associated with traditional cardiometabolic risks of adiposity and glucose homeostasis. Most consistently, increased adiposity was associated with fewer large HDL-particles. This reduction in large HDL-particles appears distinct from, and potentially additive with, the inactivity-associated reductions in small HDL-particles, which together may culminate in significant pro-atherogenic effects.

These findings are consistent with recognition that HDL-particles have heterogeneous functions and composition [12, 24]. Typically, both large and small HDL-particles participate in reverse cholesterol transport, the major driver of the inverse relationship between HDL-particles and cardiometabolic disease [12]. Also, HDL-particles exert vasodilatory and anti-thrombotic effects, primarily mediated by large HDL-particles [24]. Additionally, protein-rich small HDL mediate anti-inflammatory and anti-oxidative functions via transport of proteins with anti-inflammatory and immunomodulatory functions [24]. However, in the setting of increased inflammation, such as in active RA, such proteins are replaced with pro-inflammatory and acute phase proteins [12, 14]. Furthermore, HDL transport of inflammatory proteins occurs at the expense of apolipoproteins resulting in less HDL-mediated reverse cholesterol transport [13]. Exacerbating these issues, inflammation increases HDL-particle catabolism and renal clearance resulting in fewer HDL-particles [12, 24].

The relationship between LDL-particles and RA is just as complex. As compared to controls, patients with RA had fewer total and small LDL-particles, resulting in a larger mean LDL-size. The association of excess CVD with lower LDL concentrations has been described as “the lipid paradox of RA;” fewer small LDL particles continues to support a paradoxical relationship. RA-associated inflammation increases cholesterol catabolism such that small, cholesteryl ester (CE)-laden particles, transfer CE to the liver at increased rates, resulting in fewer small CE containing LDL and lower LDL-C [13]. However, in our RA cohort, increased inflammatory cytokines were not associated with fewer small LDL-particles and larger mean LDL-sizes; instead, they were associated with smaller mean LDL-particle sizes. Thus, it appears that the RA-associated LDL profile is not mediated entirely by inflammation. Additionally, as traditional cardiometabolic risk factors were associated with more total and small LDL-particles, it appears the RA-associated LDL profile (fewer total and small LDL) is not mediated by adiposity or insulin resistance.

Interestingly, both less time exercising and more basal or minimal physical activity were associated with fewer small LDL-particles and larger mean LDL-sizes. Further, the effects of minimal physical activity on LDL-particles were independent of exercise time. It may be that less physically active patients are those with higher disease activity and concomitantly have the typical dyslipidemia that is observed in active rheumatic disease (i.e., decreased small LDL- and HDL-particles and normal total and LDL-cholesterol levels). However, rather than larger LDL sizes, smaller LDL sizes were associated with increased concentrations of several inflammatory cytokines. Consequently, rather than concluding that physical activity worsens LDL profiles, it appears more likely that patients who were more active tended to have a reversal of the typical inflammatory dyslipidemia that is observed in RA patients [10, 13, 14]. These lipoprotein changes may be mediated by anti-inflammatory effects of exercise [25–29]. Similarly, lipoprotein changes are observed in RA patients who have been treated with anti-TNF-alpha agents, IL-6 receptor antagonists and JAK inhibitors [13, 14, 30, 31]. Concomitant HDL function improvements suggest these shifts are atheroprotective [13, 31].

We recognize that this study has limitations. The cross-sectional nature makes it impossible to determine causality. A small number of subjects was used for this study, therefore the results may be considered preliminary and larger studies are needed to confirm these results. Also, we recognize that multiple statistical analyses were performed without correction for multiple testing. However, we focused on associations that were consistent across multiple similar, yet independent, measures, reported strengths of associations and ranges of significance such that the reader can consider these in interpretation of the findings.

## Conclusions

In this RA cohort with mild to moderate disease activity, we found lipoprotein profiles similar to those previously reported: there were fewer total and small LDL particles in the setting of larger HDL particles, consistent with the RA lipoprotein profile. We identified intriguing associations for basal or minimal physical activity and exercise time with lipoprotein parameters suggestive that a large part of the RA lipid profile is mediated by lack of physical activity. These findings call for intervention studies in RA evaluating the impact of reduced sedentary time, in the setting of increased disability and pain, on lipoproteins and overall cardiometabolic health; they also call for research designed to understand the underlying mechanisms of these observed effects.

## Abbreviations

BMI: Body mass index
CE: Cholesterol ester
CVD: Cardiovascular disease
DAS-28: Disease activity score
DAS_ESR_-28: Disease activity score with 28-joint count using erythrocyte sedimentation rate
ESR: Erythroctye sedimentation rate
HAQ-DI: Health assessment questionnaire-disability index
HDL: high density lipoprotein
HOMA: Homeostasis model assessment
hsCRP: High sensitivity C-reactive protein
IL: Interleukin
LDL: Low density lipoprotein
RA: Rheumatoid arthritis
TNF-α: Tumor necrosis factor-alpha
VAS: Visual analogue scale
